# Influenza vaccination program for medical workers

**DOI:** 10.4103/1817-1737.69123

**Published:** 2010

**Authors:** Viroj Wiwanitkit

**Affiliations:** *Wiwanitkit House, Bangkhae, Bangkok, Thailand – 10160, Bangkok. E-mail: wviroj@yahoo.com*

Sir,

I read the recent publication on influenza vaccination program for medical workers with a great interest.[1] Al-Otaibi *et al*. reported that “engaging nurses from all departments may have contributed to this high influenza vaccine coverage, especially among nurses”.[1] I would like to share ideas on this specific issue. First, the basic question to be discussed is “forced” or “not forced” vaccination for medical workers. In some countries, in the pandemic influenza status, medical workers are forced to get influenza vaccine to assure that they will be safe for getting disease from the patients and they will not transmit the disease to the patients on the other hand. Second, it is interesting that although medical workers have higher knowledge on disease, their decision to get or not to get vaccine seems to be based on emotional factor. Finally, “what is the best technique to promote the highest rate of vaccination?” is the interesting question. A recent report by Talbot et al. stated that “Vaccination programs that emphasized accountability to the highest levels of the organization, provided weekend access to vaccination, and used train-the-trainer programs had higher vaccination coverage”.[2]
